# Optimising care for patients with cognitive impairment and dementia following hip fracture

**DOI:** 10.1007/s00391-017-1224-4

**Published:** 2017-03-31

**Authors:** Nigel Gill, Simon Hammond, Jane Cross, Toby Smith, Nigel Lambert, Chris Fox

**Affiliations:** 1 Norwich Medical School, University of East Anglia, Earlham Road, NR4 7TJ Norwich, Norfolk UK; 2 School of Health Sciences, University of East Anglia, NR4 7TJ Norwich, UK; 3 Hellesdon Hospital, Norfolk and Suffolk NHS Foundation Trust, NR6 5BE Norwich, Norfolk UK

**Keywords:** Healthcare system, Length of stay, Delirium, Acute care, Rehabilitation, Gesundheitssystem, Aufenthaltsdauer, Delir, Akutversorgung, Rehabilitation

## Abstract

The global shift in demographics towards aging populations is leading to a commensurate increase in age-related disease and frailty. It is essential to optimise health services to meet current needs and prepare for anticipated future demands. This paper explores issues impacting on people living with cognitive impairment and/or dementia who experience a hip fracture and are cared for in acute settings. This is important given the high mortality and morbidity associated with this population. Given the current insufficiency of clear evidence on optimum rehabilitation of this patient group, this paper explored three key themes namely: recognition of cognitive impairment, response by way of training and education of staff to optimise care for this patient group and review of the importance of outcomes measures. Whilst there is currently insufficient evidence to draw conclusions about the optimal ways of caring for patients living with dementia following hip fracture, this paper concludes that future research should improve understanding of healthcare staff education to improve the outcomes for this important group of patients.

## Zusammenfassung

Die globale Verschiebung hin zu einer alternden Bevölkerung führt zu einem entsprechenden Anstieg altersbedingter Erkrankungen und Frailty. Es ist essenziell, die Gesundheitsversorgung zu optimieren, um den derzeitigen Bedarf zu decken und sich auf zu erwartende künftige Anforderungen vorzubereiten. Dieser Beitrag untersucht die Probleme und deren Auswirkungen auf Menschen mit kognitiven Störungen und/oder Demenz, die eine Hüftfraktur erlitten haben und im Rahmen der Akutversorgung behandelt werden. Dies ist hinsichtlich der hohen Mortalität und Morbidität, die mit dieser Population assoziiert ist, von großer Bedeutung. Aufgrund des derzeitigen Mangels an klarer Evidenz bezüglich einer optimalen Rehabilitation dieser Patientengruppe, wurden in dieser Arbeit 3 Hauptthemen untersucht: Erkennung einer kognitiven Störung, die Resonanz durch Fort- und Weiterbildung des Gesundheitspersonals, um die Versorgung dieser Patientengruppe zu optimieren, sowie die Überprüfung der Bedeutsamkeit von Ergebnisparametern. Während die aktuelle Evidenzlage nicht ausreicht, um Rückschlüsse bezüglich der optimalen Vorgehensweise bei der Versorgung von dementen Patienten nach Hüftfraktur zu ziehen, kommt dieser Beitrag zu dem Schluss, dass zukünftige Untersuchungen die Bedeutung der Ausbildung des Gesundheitspersonals deutlich machen sollten, um die Ergebnisse für diese wichtige Patientengruppe zu verbessern.

## Background

The commonest causes of cognitive impairment in the elderly are dementia, delirium or a combined presentation [1]. Admission to hospital for surgical treatment of hip fractures is likely to be associated with some form of cognitive impairment, which may be precipitant to the trauma or prolonging to the hospital stay and frequently interdependent. In this context, dementia relates to progressive cognitive decline and cognitive impairment and includes potentially temporary or reversible states that include delirium. The incidence of dementia is reaching epidemic proportions [2] with 46 million people living with dementia worldwide and this is estimated to increase to 131.5 million by 2050 [3, 4]. Dementia is more prevalent with age, increasing frailty and with comorbid conditions, which results in complex health and social needs for these people. It is recognised [5] that hospitalisation has adverse effects for patients living with dementia [6]. Their differing needs resulting from changes in their memory, orientation, comprehension, calculation, learning capability, language and judgment [6] are often unrecognised and unaddressed. Regardless of the reason for hospitalisation people living with dementia are reported to have half the survival time of those without dementia following acute admission [7] and a functional outcome which is 64% worse [8]. More specifically outcomes following fractures are poorer when compared to a cognitively intact cohort [9, 10].

Hip fracture is a common orthopedic injury amongst older adults and accounts for 87% of the cost of all fragility fractures [11]. It is estimated that 95% of hip fractures are due to falls [12]. The annual prevalence of hip fractures globally is expected to reach 4.5 million by 2050 [13]. The economic implications of hip fractures are significant, with 32 × 10^9^ euros per year in Europe and 20 × 10^9^ US dollars per year in the United States [14] with costs for rehabilitation being a significant part of this [15, 16]. In the United Kingdom (UK), over 70,000 people suffer a hip fracture each year resulting in 2 × 10^9^ pounds costs for health and social care [17]. It is estimated that the annual number of fractures in the EU will rise from 3.5 million in 2010 to 4.5 million in 2025, an increase of 28%. Approximately 40% of those with hip fractures have a diagnosis of dementia [18, 19]. An individual with dementia is up to three times more likely to suffer a hip fracture than someone who is cognitively intact [20, 21]. Reports from the UK suggest an estimated 0.4 × 10^9^ pounds sterling more is spent on caring for hip fracture patients living with dementia than those deemed psychiatrically well [22] and this is predicted to rise by 30% in the next 10 years [23]. A Cochrane review published in 2015 found insufficient evidence to draw conclusions about the optimal methods for caring for patients living with dementia and experiencing a hip fracture [10].

In this article we undertake a narrative review of the themes related to rehabilitation of this patient group based on work from our perfected research programme (www.perfected.ac.uk), to aid clarity and inform about future research.

## Recognition of the need

There has been debate about whether people with cognitive impairment (from whatever cause) should be seen as different to those considered cognitively intact. Care delivery to patients with dementia in acute hospitals has advanced under the spotlight that was attracted by examples of detrimental care [21, 24]. The evidence shows that cognitive impairments must be actively identified for appropriate management to be undertaken [25–28]. Studies [26, 28, 29] have shown that cognitive impairment is under-detected, and that lack of detection is linked to poorer care. There is a lack of awareness among hospital staff of the importance of identification coupled with inadequate knowledge and skills to detect cognitive impairment [26, 28, 29]. Dementia alone is rarely the reason for their admission although this may well be the underlying cause.

Several studies [30, 31] showed high degrees of variability in hospital practice regarding whether assessment of cognitive impairment is done and if so which tools are used. This variability appears to be for different reasons [31, 32]. The UK has attempted to standardise care and hospitals must now produce a dementia strategy [33, 34]. This has led to a greater awareness regarding how physical, visual, acoustic and psychosocial environments operate to make wards more ‘dementia friendly’; however, access to specialist care and facilities may only be available to patients with an established dementia diagnosis. Patients lacking a formal diagnosis or experiencing cognitive impairment due to other causes will be denied access. It is therefore imperative that these initiatives become ubiquitous as demonstrated in approaches towards frailty; here patients are seen as frail until shown otherwise [35]. This has caveats, such as the need to balance deprivation of liberties, challenge ageist narratives and steer clear of phenotyping all elderly people as frail or cognitively vulnerable; however, a proactive stance toward embedding dementia sensitive practices and resources offers ways to alter care aspirations amongst healthcare staff. We are not saying that ‘dementia friendly’ initiatives are undervalued, in fact their remit needs to transcend geriatric medicine. Such environments and care ethos could substantially benefit all elderly patients, not just those with a formal diagnosis of dementia. The extension of dementia strategies could be positive as all patients may benefit from better designed hospital environments and therapeutic interactions tailored to individual needs. The demographic shift toward older populations will lead to a larger proportion of frail hospital patients in whom even relatively minor physiological and/or psychological stressors may be associated with adverse health outcomes [36].

The widespread ‘re-engineering’ of hospital practice may not be feasible. Instead, preparation must be made to ‘future-proof’ hospitals and services to cope with these anticipated changes. Calls for moves away from a ‘silo mentality’ find traction in relation to improving hospital care for people living with cognitive impairment and there is a need to widen the remit of ‘dementia friendly’ to inform healthcare design needs [37].

## Response, training and education

Given that many healthcare staff in acute hospital settings are not currently equipped to care for people with cognitive impairment [38], it is evident that education is critical to change [38–43].

Research reports that many staff prioritise caring for the physical health needs of patients [41]. Managing behaviours associated with dementia may not be seen as part of their role and this may lead to patients with purely physical health needs being given greater priority than those also exhibiting cognitive impairment [44]. The role of providing extra support to people with cognitive impairment during a hospital admission often falls to healthcare assistants, for example increased monitoring if patients are ‘wandersome’.

Education and training should be easily accessible and organisation led [45, 46] but must go beyond educating individuals in order to facilitate positive organisational change [47]; however, this alone will not sustain a change in the culture of care [47, 48]. Training could be directed at staff at an emotional and intellectual level [48], supporting empathy and focus on person-centred approaches [42, 48, 49]. Dementia training should be targeted at wards and also at management if issues such as poor staffing and pressure to complete medical tasks quickly are to be addressed. This may overcome a strategy for an efficiency-driven organisation [50].

The literature reports a number of implementation barriers [41, 51, 52] including lack of time or resources, lack of confidence and competence in identifying the needs of people living with dementia and dealing with behaviour that challenges and an inability to identify levels of cognitive impairment accurately and negative attributions made towards people with dementia. Smythe et al. [52] described staff members who attributed the challenges associated with caring as being the fault of the person living with dementia.

Patients with cognitive impairment admitted to orthopedic/orthogeriatric wards after hip fracture present complications that require additional skills from healthcare teams, for example, considering the consequent reduced mobility after hip fracture. A patient with dementia who likes to walk as a means of interacting with their environment may need to remain immobile at first to reduce the risk of falling and further injury. Reducing their mobility by restricting them to their bed or chair can result in increased agitation and aggression if not managed skilfully [53]. Using antipsychotics or hypnotics to manage such behaviour may induce or exacerbate delirium. The allocation of a ‘special’ (member of staff employed to remain in close proximity to try to maintain safety) can lead to an increased sense of paranoia as the patient may not comprehend the purpose of someone remaining so close for long periods of time.

Rehabilitation following hip fracture surgery is regarded as essential for patients to regain the mobility they had prior to the fracture; however, rehabilitation and most forms of movement for these patients can be painful. This poses unique communication problems as these patients can struggle to understand the need for rehabilitation therapies and if their therapy is painful, they may interpret this as abuse. This is complicated by the difficulty in assessing what pain relief such patients require.

There is some evidence for dementia training programmes [54, 55] and highlights training should be directly applicable to ward environments and should increase the confidence of healthcare staff [56, 57]. Staff training should be tailored for general hospital wards, improve staff confidence, shift perceptions about the etiology of challenging behaviour and help staff view their role as addressing the needs of people living with dementia.

## Redefine outcome measures

Outcome measures in the research context are defined for specific purposes related to the acquisition of knowledge, in clinical practice they can be used more formatively to shape activity or define organisational goals. Outcome measures are used to strengthen healthcare management by identifying the type and level of achievement that is desired [58]. They are considered useful in the public sector as they focus the attention of government agencies, such as healthcare providers, and in the private sector to motivate the workforce toward corporate objectives.

The hospital length of stay (LoS) is widely used as an outcome measure. In hip fractures, a hospital that achieves shorter LoS is considered ‘successful’. Financial reward is provided for hospitals with shorter admission periods. Some international insurance based/payee healthcare systems use similar drivers to minimise hospital stay. Although LoS may be a measure which is intuitively appealing it is not without its drawbacks. An assumption is made that each day in hospital costs the same as the next when in fact the most expensive days are those when a patient is first admitted and undergoes the most active investigations and treatment and it may be more expensive to discharge a patient early if they are subsequently readmitted and there may be a financial penalty for a ‘failed discharge’. In many ways LoS is the body mass index (BMI) of modern medicine. It is simplistic and readily available, as opposed to being accurate and indicative of quality of care. In addition, the use of such overly simple and blunt measures as LoS risks ‘metric manipulation’ [59], a situation where managers may manipulate target and performance measures for organisational gain. To reduce the LoS in acute hospitals, some patients may be relocated to rehabilitation units (or community hospitals) for intensive occupational therapy and physiotherapy. Such units are seen as more effective at providing rehabilitation and physically safer [5]. Thus, for acute hospitals with ready access to such rehabilitation units, LoS statistics can appear to be as low as 2–3 days postoperation; however, where access to such units is limited, LoS is longer and thus not a true measure of treatment effectiveness or quality. Further complication occurs if the rehabilitation unit is part of the same acute hospital as this counts as part of the same LoS making comparison difficult in terms of quality and efficiency between different providers. Discharge from acute hospitals can be a lengthy and time-consuming process. Medically fit patients can wait many weeks, even months, to be discharged to a secure domicile.

A more treatment-orientated and fairer indication of hospital efficiency may be length of time before patients are medically fit for discharge; however, this measure is not without problems; a patient is deemed ‘medically fit’ when the treating consultant or their deputy decides that acute hospital treatment is no longer required [60]. It does not indicate that the patient has returned to full health as they may still require rehabilitation.

Initiatives to prevent, identify and treat delirium are most welcome as are initiatives that identify other treatable conditions that predispose to falls. Tools such as the Confusion Assessment Method are available but their use is reported as patchy due to the complex nature of the assessment and the level of skill required to undertake testing [61]. Initial costs for setting up such initiatives is also an issue but account also needs to be taken of the longer-term savings from reducing delirium and falls. There is a paucity of health economic studies to encourage providers to expand such initiatives.

## Conclusion

Given shifts in population demographics, the optimisation of rehabilitation for hip fracture patients with cognitive impairment is of high importance due to the expected increase in incidence, economic cost and personal cost to individuals and their families. Currently there is insufficient evidence to draw conclusions about the best methods of caring for patients living with dementia following a hip fracture [10]. To develop optimum methods of rehabilitation, it is suggested that the following priorities should be addressed: to further refine tools that enable the identification of cognitive impairment in this group of patients, staff education to develop specialised skills and understanding of their importance once cognitive impairment has been identified, redefining outcome measures and recognising that these are important in shaping activity and defining organisational goals.

## References

[1] Jackson TA (2016). Undiagnosed long-term cognitive impairment in acutely hospitalised older medical patients with delirium: a prospective cohort study. Age Ageing.

[2] World Health Organization (2015). The epidemiology and impact of dementia current state and future trends.

[3] Ali G-C (2015). World AlzheImer Report 2015 – the global prevalence of dementia.

[4] Prince M, Ali G-C, Guerchet M, Prina MA, Albanese E, Wu YT (2016). Recent global trends in the prevalence and incidence of dementia, and survival with dementia. Alzheimers Res Ther.

[5] Boaden A (2016). Fix dementia care: hospitals.

[6] Crowther GJE, Bennett MI, Holmes JD (2017). How well are the diagnosis and symptoms of dementia recorded in older patients admitted to hospital?. Age Ageing.

[7] Sampson EL (2013). Survival of people with dementia after unplanned acute hospital admission: a prospective cohort study. Int J Geriatr Psychiatry.

[8] Hartley P, Gibbins N, Saunders A, Alexander K, Conroy E, Dixon R, Lang J, Luckett J, Luddington T, Romero-Ortuno R (2017) The association between cognitive impairment and functional outcome in hospitalised older patients: a systematic review and meta-analysis. Age Ageing:. doi:10.1093/ageing/afx007 10.1093/ageing/afx007 28119313

[9] Scandol JP, Toson B, Close JC (2013). Fall-related hip fracture hospitalisations and the prevalence of dementia within older people in New South Wales, Australia: an analysis of linked data. Injury.

[10] Smith TO, Hameed YA, Cross JL, Henderson C, Sahota O, Fox C (2015). Enhanced rehabilitation and care models for adults with dementia following hip fracture surgery. Cochrane Database Syst Rev.

[11] Sahota O, Currie C (2008). Hip fracture care: all change. Age Ageing.

[12] Mitchell R (2016). Hip fracture and the influence of dementia on health outcomes and access to hospital-based rehabilitation for older individuals. Disabil Rehabil.

[13] Gullberg B, Johnell O, Kanis JA (1997). World-wide projections for hip fracture. Osteoporos Int.

[14] International Osteoporosis Foundation (2016). Capture the fracture: health economics.

[15] Zielinski SM (2014). The societal costs of femoral neck fracture patients treated with internal fixation. Osteoporos Int.

[16] Burgers PT (2016). Total medical costs of treating femoral neck fracture patients with hemi- or total hip arthroplasty: a cost analysis of a multicenter prospective study. Osteoporos Int.

[17] Chamberlain M, Pugh H (2015). Improving inpatient care with the introduction of a hip fracture pathway. BMJ Qual Improv Rep.

[18] Seitz DP, Adunuri N, Gill SS, Rochon PA (2011). Prevalence of dementia and cognitive impairment among older adults with hip fracture. J Am Med Dir Assoc.

[19] Boulton C (2016). National Hip Fracture Database annual report 2016.

[20] Friedman SM (2010). Dementia and hip fractures: development of a pathogenic framework for understanding and studying risk. Geriatr Orthop Surg Rehabil.

[21] Francis R (2013). Report of the Mid Staffordshire NHS Foundation Trust Public Inquiry.

[22] Bourn J (2007). Improving services and support for people with dementia.

[23] National Institute of Clinical Excellence (2011). Hip fracture: management. Clinical guideline [CG124].

[24] Manthorpe J (2015). The abuse, neglect and mistreatment of older people with dementia in care homes and hospitals in England: the potential for secondary data analysis: innovative practice. Dementia (London).

[25] Timmons S (2015). Dementia in older people admitted to hospital: a regional multi-hospital observational study of prevalence, associations and case recognition. Age Ageing.

[26] Briggs R (2017). Dementia in the acute hospital: the prevalence and clinical outcomes of acutely unwell patients with dementia. QJM.

[27] Travers C (2013). Prospective observational study of dementia and delirium in the acute hospital setting. Intern Med J.

[28] Partridge JS (2014). The prevalence and impact of undiagnosed cognitive impairment in older vascular surgical patients. J Vasc Surg.

[29] Sampson EL (2009). Dementia in the acute hospital: prospective cohort study of prevalence and mortality. Br J Psychiatry.

[30] Jenkin RPL, Musonda P, MacLullich AMJ, Myint PK, Davis DHJ (2014). Specialty experience in geriatric medicine is associated with a small increase in knowledge of delirium. Age Ageing.

[31] Royal College of Psychiatrists (2016). The national audit of dementia.

[32] Hammond SP, Cross JL, Poland FM, Patel M, Penhale B, Smith TO, Fox C (2016). Freedom of Information Act: scalpel or just a sharp knife?. J Med Ethics.

[33] United Kingdom Department of Health (ed) (2015) 2010 to 2015 government policy: dementia

[34] United Kingdom Department of Health (ed) (2009) Living well with dementia: a national dementia strategy

[35] Lin SY (2017). ‘Dementia-friendly communities’ and being dementia friendly in healthcare settings. Curr Opin Psychiatry.

[36] Harrision JK, Clegg A, Conroy SP, Young J (2015). Managing frailty as a long-term condition. Age Ageing.

[37] Prince M, Comas-Herrera A, Kemp M, Guerchet M, Karagiannidou M (2016). The World Alzheimer Report 2016.

[38] Sampson EL et al (2016) Improving the care of people with dementia in general hospitals: evaluation of a whole-system train-the-trainer model. Int Psychogeriatr:. doi:10.1017/s1041610216002222 10.1017/S1041610216002222 27998325

[39] Eriksson C, Saveman BI (2002). Nurses’ experiences of abusive/non-abusive caring for demented patients in acute care settings. Scand J Caring Sci.

[40] Cowdell F (2013). “that’s how we do it … we treat them all the same”: An exploration of the experiences of patients, lay carers and health and social care staff of the care received by older people with dementia in acute hospital settings.

[41] Borbasi S (2006). Health professionals’ perspectives of providing care to people with dementia in the acute setting: Toward better practice. Geriatr Nurs.

[42] Nolan L (2007). Caring for people with dementia in the acute setting: a study of nurses’ views. Br J Nurs.

[43] Rhynas SJ (2010). ‘Forgotten shopping, lost keys and hearts which forget to beat’: An exploration of nurses’ conceptualisation of dementia.

[44] Calnan M, Tadd W, Calnan S, Hillman A, Read S, Bayer A (2013). ‘“I often worry about the older person being in that system”: exploring the key influences on the provision of dignified care for older people in acute hospitals. Ageing Soc.

[45] Jones J, Borbasi S, Nankivell A, Lockwood C (2006). Dementia related aggression in the acute sector: is a Code Black really the answer?. Contemp Nurse.

[46] Deeks LS, Cooper GM, Draper B, Kurrle S, Gibson DM (2016). Dementia, medication and transitions of care. Res Social Adm Pharm.

[47] Moyle W (2011). Care staff attitudes and experiences of working with older people with dementia. Australas J Ageing.

[48] Cowdell F (2010). The care of older people with dementia in acute hospitals. Int J Older People Nurs.

[49] Nolan L (2006). Caring connections with older persons with dementia in an acute hospital setting – a hermeneutic interpretation of the staff nurse’s experience. Int J Older People Nurs.

[50] Nilsson A, Rasmussen BH, Edvardsson D (2013). Falling behind: a substantive theory of care for older people with cognitive impairment in acute settings. J Clin Nurs.

[51] Atkin K, Holmes J, Martin C (2005). Provision of care for older people with co-morbid mental illness in general hospitals: general nurses’ perceptions of their training needs. Int J Geriatr Psychiatry.

[52] Smythe A (2014). Evaluation of dementia training for staff in acute hospital settings. Nurs Older People.

[53] Agens JE (2010). Chemical and physical restraint use in the older person. Br J Med Pract.

[54] Elvish R, Burrow S, Cawley R, Harney K, Graham P, Pilling M, Gregory J, Roach P, Fossey J, Keady J (2014). ‘Getting to Know Me’: the development and evaluation of a training programme for enhancing skills in the care of people with dementia in general hospital settings. Aging Ment Health.

[55] Galvin JE, Kuntemeier B, Al-Hammadi N, Germino J, Murphy-White M, McGillick J (2010). “Dementia-friendly hospitals: care not crisis”: an educational program designed to improve the care of the hospitalized patient with dementia. Alzheimer Dis Assoc Disord.

[56] Turner A, Eccles FJR, Elvish R, Simpson J, Keady J (2015). The experience of caring for patients with dementia within a general hospital setting: a meta-synthesis of the qualitative literature. Aging Ment Health.

[57] Chater K, Hughes N (2013). Strategies to deliver dementia training and education in the acute hospital setting. J Res Nurs.

[58] Hauck K, Street A (2007). Do targets matter? A comparison of English and Welsh National Health priorities. Health Econ.

[59] Fisher C, Downes B (2006). Performance management and metric manipulation in the public sector.

[60] UK Legislation (2014). Care Act 2014.

[61] Filinson R (2016). Adoption of delirium assessment in the acute care setting: a tale of two hospitals. Best Pract Ment Health.

